# Expanding Knowledge About Implementation of Pre-exposure Prophylaxis (PrEP): A Methodological Review

**DOI:** 10.1007/s10461-019-02577-7

**Published:** 2019-07-10

**Authors:** Rogério M. Pinto, Ashley Lacombe-Duncan, Emma Sophia Kay, Kathryn R. Berringer

**Affiliations:** 1 University of Michigan, School of Social Work, Office 2850, 1080 South University, Ann Arbor, MI 48109 USA; 2 University of Michigan, Anthropology, Ann Arbor, MI USA

**Keywords:** PrEP implementation methods, PrEP methodological review, HIV prevention, Continuum of care

## Abstract

Methodological limitations in PrEP implementation studies may explain why PrEP implementation is lagging. This methodological review provides a description and critique of the methods used to identify barriers to PrEP implementation in the United States (2007–18). For each selected article, we provide: (1) research questions; (2) measures; (3) design; (4) sample (size and type); and (5) theoretical orientation. Among 79 articles which identified knowledge, attitudes, and behavioral and social/structural barriers to PrEP implementation, 51 (65%) were quantitative; 25 (32%) qualitative; and 3 (4%) were mixed-methods; overall, just one-half described a conceptual approach. About two-thirds of articles were conducted with patients and one-third with healthcare providers. Our review reveals a paucity of longitudinal, mixed-methods, and ethnographic/observational research and guiding theoretical frameworks; thus, the applicability of results are limited. We recommend that interventions aimed at PrEP implementation address barriers situated at multiple ecological domains, and thus improve PrEP access, uptake, and adherence.

## Introduction

At the end of 2015, the United States Centers for Disease Control and Prevention (CDC) estimated that 1,122,900 persons in the United States (US) were living with HIV; of these, 38,500 new infections occurred in 2015 alone [1]. In 2012, the US Food and Drug Administration (FDA) approved HIV pre-exposure prophylaxis (PrEP)—Truvada™ [Emtricitabine/Tenofovir Disoproxil Fumarate (TDF/FTC)]—as a daily dosing strategy to reduce the risk of HIV infection for people most exposed. Research shows a risk reduction by 73% among adult men who have sex with men (MSM) and transgender women who take PrEP 90% of the time [2]; and even greater efficacy (up to 99%) for people with higher rates of PrEP adherence [3, 4]. Though PrEP reduces risk for HIV infection, only 56,600 Latino, African American, and White people are estimated to be taking PrEP; even though an estimated 1.1 million people from these groups may benefit based on CDC clinical guidelines—this amounts to only about 5% of all people in the US who could benefit from PrEP taking it [5, 6].

In a comprehensive review of the literature, our team identified 30 barriers to PrEP implementation—steps patients and health providers must take in order to navigate healthcare systems and to ascertain access, delivery, and adherence to PrEP [7]. As from our original review, herein, the steps patients and providers must take to follow policies governing access to PrEP and to navigate healthcare systems are referred to as “PrEP implementation.” Our conceptualization reflects the definition of implementation research as the “study of processes and strategies that move, or integrate, evidence-based effective treatments [in this case PrEP] into routine use, in usual care settings.” ([8], p. 27) The review included research, between 2007 and 2017, in the fields of medicine, nursing, social work, and public health. We identified barriers across four ecological domains individual (patient), relationship (patient-service provider), community, and policy [9]. Among cognitive barriers, those affecting patients and providers included lack of knowledge about, and negative attitudes toward PrEP. Healthcare-level barriers included lack of communication about, funding for, and access to PrEP. The “purview paradox” was a key barrier—HIV specialists trained to provide PrEP often do not tend to HIV-negative patients, while primary care physicians, who often see uninfected patients, are often not trained to provide PrEP. PrEP stigma and HIV stigma, transphobia and homophobia, sexism, and racism are also major barriers to PrEP implementation, contributing to disparities across sexual orientation, gender identity, and racial/ethnic background.

In order to decrease the rate of HIV infection, interventions to scale up PrEP will need to address identified barriers at multiple ecological levels. However, in the past decade, interventions that have been proposed to break PrEP implementation barriers have often been limited to one ecological level or another (e.g., individual or community). The failure to consider interventions targeting multiple ecological levels simultaneously may explain partly why PrEP implementation is lagging [7]. However, this failure may also be due to methodological limitations of PrEP implementation studies, as evidenced in our systematic review where we saw that studies tended to focus on one level (e.g., patient-level) while making recommendations on another (e.g., provider-level) without supporting data [7]. We also found few published intervention studies to guide implementation, perhaps indicative of timing (e.g., early in PrEP implementation). Based on our systematic review of the PrEP implementation literature, we hypothesize that the methods used thus far have also been limited in several ways beyond sample characteristics (e.g. patient versus provider) and study design (e.g., exploratory versus intervention). Questions remain about the extent to which the methods used to identify barriers to PrEP implementation have progressed since PrEP became a major HIV prevention strategy and how research can be conducted to better inform advancements in PrEP implementation science.

Therefore, we have conducted an evaluation of the methods used thus far to identify barriers to PrEP implementation. We organized the current methodological review chronologically, and, for each article reviewed, we provide a summary of: (1) key elements of research questions; (2) measures; (3) research design; (4) sample (size and type); and (5) theoretical orientation. We describe how methods to study PrEP implementation have evolved over time, and we make recommendations about how to build on these methods to better capture PrEP implementation barriers and corresponding solutions as we move forward.

## Methodological Review: Conceptual Approach

This methodological review is grounded in Whittemore et al. [10] model for integrative reviews and Munn et al. [11] typology/guidance for systematic reviews in the medical and health sciences. The integrative model and the typology guided us in our choice of a specific and clear set of inclusion/exclusion criteria for selection of articles, followed by a comprehensive search of published articles within a well-defined time period.

This methodological review builds on a systematic review that we published in 2018, and which included an examination of barriers to PrEP implementation published between 2007 and 2017 [7]. The current methodological review extends the original one to include 79 articles published between 2007 and 2018. The key goal of the original review was to identify barriers to PrEP implementation in the US. We were guided by a socioecological perspective [9] suggesting that barriers to PrEP implementation reside within different domains of reference: Individual and Relationships (patients and care providers); and Community and Policy Domains (policies governing HIV-prevention efforts, and both healthcare systems and agency settings guidelines). This approach in the context of PrEP implementation recognizes the roles of both patients and healthcare providers embedded within healthcare systems of all sizes who are required to follow multiple policies and guidelines [12]. In this case, these policies and guidelines refer to those regarding PrEP implementation—steps to navigate healthcare systems and which facilitate patient access and adherence to PrEP. By maintaining a socioecological approach to this methodological review, we continue to attend to the ways in which PrEP study methods address the holistic contexts within which PrEP implementation occurs.

## Methods

### Procedures for Article Selection: Inclusion and Exclusion Criteria

For the current methodological review, we updated the time period (2007–2017) we used for the original systematic review in order to include articles published in 2018. For the present methodological review, we changed inclusion and exclusion criteria slightly so as to include only articles containing a clear description of methods. In summary, we selected articles published between January 2007 and December 2018, a time period that included the development of the HIV continuum of care and high-impact prevention approach (treatment as prevention), the surge of evidence of PrEP effectiveness from large-scale clinical trials [2, 13, 14], the subsequent FDA approval of PrEP for service settings [15], and, more recently, the confirmation that daily PrEP use is safe [16, 17].

We used the University of Michigan’s ArticlesPlus, a comprehensive database of peer-reviewed clinical and academic journals in medicine, public health, social work, nursing, pharmacy, and law, to conduct our literature search. Our combination of search terms, including truncation operators (*) as follows:

Subject Terms: (HIV OR HIV/AIDS OR AIDS) AND Title: (PrEP OR “Pre-Exposure Prophylaxis”) OR [(antiretroviral* OR pharmaceutical*) AND prevent*)] AND All Fields: [(worker* OR practitioner* OR provider*) AND (linkage* OR linking OR referral* OR implementation OR uptake)].

Our initial search (January 2007–June 2018) yielded 196 articles that [1] described implementation of PrEP programs for HIV prevention, and [2] focused on HIV service providers, medical, and social and public health service providers in agency settings in the US, and patients. We focused exclusively on the US because PrEP-related implementation policies and practices may differ profoundly across the globe. The inclusion criteria used to search articles published in 2018 were the same used for our published systematic review about barriers to PrEP implementation [7].

In order to include articles published through the end of the 2018, we attempted to update our search in January 2019. Unfortunately, in the interim the database system, *ArticlesPlus,* on which we had conducted our original search was discontinued by the University of Michigan. Therefore, we conducted our updated search using the new University of Michigan search interface, which consolidated *ArticlesPlus* with other library search interfaces. In order to meet the parameters of this new search tool, our search terms needed to be altered slightly (to limit operators and parenthetical clauses included within search term categories). The following updated search was conducted on January 13, 2019:

[subject:HIV OR HIV/AIDS OR AIDS AND title:PrEP OR “Pre-Exposure Prophylaxis” AND all_fields:linkage* OR linking OR referral* OR implementation OR uptake] OR [subject:HIV OR HIV/AIDS OR AIDS AND title:antiretroviral* OR pharmaceutical* AND prevent* AND all_fields:linkage* OR linking OR referral* OR implementation OR uptake]

**Fig. 1:**
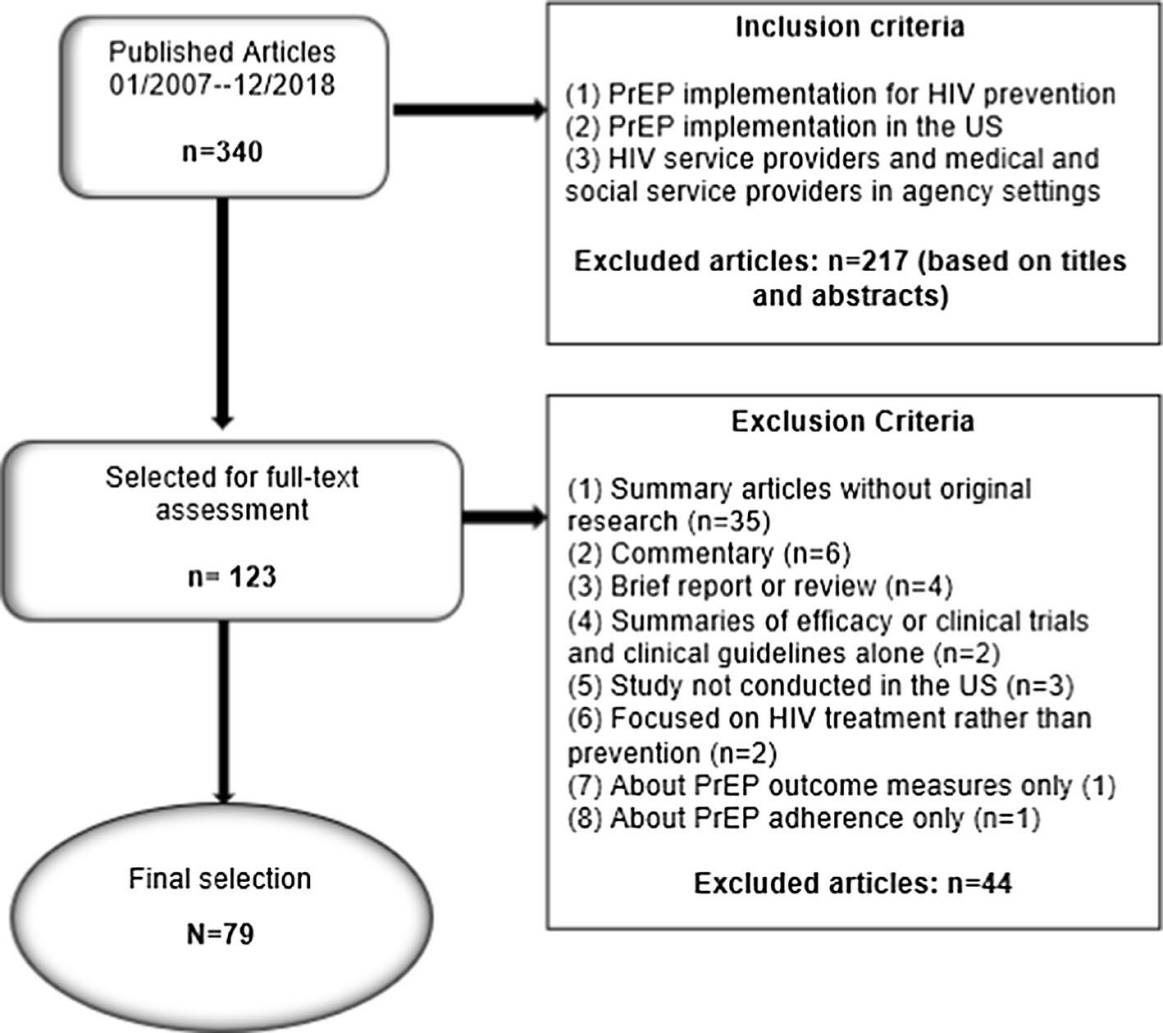
Article selection and inclusion/exclusion criteria

Compared to our original search, which yielded 196 articles between 2007 and June 2018, this consolidated search tool yielded many more results. The search generated 340 articles published between 2007 and 2018, with 144 articles published in 2018 alone. We conducted a preliminary review based on titles and abstracts, eliminating 217 articles that did not meet inclusion criteria. The remaining 123 articles were then subjected to a full-text review, at which point we were able to eliminate an additional 44 articles that did not meet inclusion criteria. The final selection (2007–2018) included 79 articles. We provide a summary of procedures for article selection in Fig. 1.

#### Summary of Exclusions

Our methodological review excluded the following types of articles: (1) summary articles without original research (e.g., systematic reviews, such as our own), (2) summaries of efficacy or clinical trials and clinical guidelines alone, (3) analysis of PrEP awareness and attitudes alone, (4) studies conducted outside of the US, (4) editorials and articles that included only a description of PrEP studies, (5) studies focused on HIV treatment rather than prevention, (6) studies about PrEP outcome measures only, (7) studies about PrEP adherence only, (8) models to determine PrEP eligibility alone, (9) epidemiological reports on PrEP and statistics alone, (10) cost-effectiveness studies alone, or (11) modelling studies alone.

### Data Extraction and Analysis

To organize and manage our library, we created an Excel spreadsheet to record key information about each publication: title; authors; journal; publication date; journal type; theoretical approach; methodological approach (i.e., qualitative, quantitative, or mixed methods); and a summary of findings.

Our analysis focused on selecting articles that identified barriers to PrEP implementation in various ecological domains and which also reflected the inclusion criteria described above. We followed the same procedures that were used in the original systematic review (for details, see [7]). In summary, to enhance rigor to the analysis, we adopted a purposive sampling strategy—explicit search terms, inclusion and exclusion criteria, and procedures for article selection [18]. We borrowed basic principles of grounded theory as we selected the final set of articles for analysis grounded in our experiences as HIV researchers and practitioners in community settings [19]. We also brought different expertise in social work and anthropology. The authors held weekly 60-min discussions to finalize the list of articles contained myriad barriers to PrEP implementation and came to 100% agreement about which articles should be included and excluded from this review.

In order to examine the methods in each article, we organized articles by type of methods used—quantitative (Table 1), qualitative (Table 2), and mixed methods approach (Table 3). For each article, we identified: (1) key elements of research questions; (2) research design; (3) sample size and type; (4) underlying theoretical approach; and (5) key measures. The authors worked individually to examine the articles, and we met six times, for meetings lasting 1–2 h, to discuss the articles and to determine the classifications based on method type and key methodological elements. During the discussions, we also decided by consensus how to present the results.

**Table 1:** PrEP implementation quantitative studies (n = 51)

Year	Article reference number	Key elements of research question (s)	Key measures	Research design	Sample (size and type)	Theoretical approach
2013	[20]	Investigate attitudes on prescribing and monitoring PrEP use	∙ PrEP knowledge/attitudes ∙ PrEP prescription rate	Cross-sectional survey	331 HIV physicians	No
	[21]	Evaluate knowledge, attitudes, perceptions, and prescribing practices	∙ PrEP knowledge/attitudes/concerns ∙ Prescribing practices	Cross-sectional survey, online	189 HIV healthcare providers (type unspecified)	No
2014	[22]	Examine perceived patient risk compensation/race on willingness to prescribe	∙ Prescribing attitudes/practices ∙ Risk compensation	Cross-sectional survey, online	102 medical school students	No
	[23]	Examine pharmacists’ experience dispensing and knowledge/concerns	∙ PrEP knowledge and concerns ∙ Experience dispensing PrEP	Cross-sectional survey	225 pharmacists	No
2015	[24]	Compare HIV and non-HIV providers on PrEP knowledge, prescription, perceived barriers	∙ PrEP knowledge/experience ∙ Perceived advantages	Cross-sectional survey	223 medical and social service providers	No
	[25]	Determine intention to prescribe PrEP	∙ PrEP knowledge and experience	Cross-sectional survey	142 medical HIV service providers	No
	[26]	Assess knowledge, practices, and perceptions about ART and PrEP	∙ PrEP prescribing practices, intentions, concerns	Cross-sectional survey, online	184 healthcare providers	Diffusion of innovation
2016	[27]	Evaluate PrEP use and retention in care outside clinical trials	∙ PrEP adherence and retention	Administrative (clinic) data, Prospective	267 PrEP patients	No
	[28]	Ascertain PrEP knowledge, attitudes, and beliefs	∙ PrEP knowledge and experience	Cross-sectional survey	403 primary care providers	Diffusion of innovation
	[29]	Explore provider experiences and practices with PrEP provision	∙ Perceived patient barriers, adherence, risk compensation ∙ PrEP feasibility	Cross-sectional survey	35 primary care physicians	No
	[30]	Examine differences in barriers and facilitators to PrEP access by race	∙ Systems-, provider-, and patient-level barriers	Cross-sectional survey	491 men who have sex with men	No
	[31]	Assess awareness of and attitudes about PrEP	∙ PrEP awareness ∙ Willingness to prescribe PrEP to ‘high risk’ groups	Cross-sectional survey	9023 physicians and nurse practitioners	No
2017	[32]	Compare perceived barriers to PrEP use by transactional sex work	∙ Hypothetical barriers to PrEP use	Cross-sectional survey	254 men who have sex with men	No
	[33]	Assess PrEP awareness, PrEP adoption, and factors associated with adoption	∙ PrEP awareness, adoption, self-rated knowledge, beliefs	Cross-sectional Survey	266 primary care physicians	No
	[34]	PrEP access after completion of a PrEP demonstration project	∙ Barriers/facilitators ∙ Perceived efficacy ∙ Access preferences ∙ Sexual behavior	Cross-sectional survey	173 former clinical trial participants	No
	[35]	Assess the relationships between potential barriers to PrEP and interest in PrEP	∙ PrEP use, awareness, and interest ∙ PrEP stigma and conspiracy beliefs	Cross-sectional survey	85 Black men and transgender women; 179 white transgender women	No
	[36]	Evaluate provider willingness to prescribe PrEP to people who use injection drugs	∙ Willingness to prescribe PrEP to potentially eligible patients	Cross-sectional survey, online	250 members of the society for academic general internists	No
	[37]	Explore characteristics associated with HIV risk perception and PrEP acceptability	∙ Sexual/reproductive health behavior ∙ Access to/engagement with support services	Cross-sectional survey	146 young women seeking healthcare	No
	[38]	Examine messages that might impact comprehension among potential PrEP users	∙ Sexual/substance abuse behavior; perceived HIV risk ∙ Health literacy/need for cognition	Cross-sectional survey	157 young people of colour	Message framing
	[39]	Understand the barriers and facilitators for PrEP willingness and uptake	∙ PrEP willingness ∙ HIV risk behaviors ∙ PrEP attitudes	Cross-sectional survey, online	687 young men who have sex with men	No
	[40]	Examine alcohol interactive toxicity beliefs and whether they impede PrEP use	∙ PrEP awareness ∙ PrEP-related interactive toxicity beliefs	Cross-sectional survey	272 men	No
	[41]	Describe PrEP use and related factors	∙ HIV/STI risk and protective behaviors	Cross-sectional survey	394 young men who have sex with men	No
	[42]	Assess history of PrEP use, indications for PrEP use, and access to healthcare	∙ PrEP utilization	Cross-sectional survey, online	2297 young men who have sex with men	No
	[43]	Examine intention to prescribe, and actual prescription of, PrEP	∙ Intentions to prescribe PrEP ∙ Actual prescription of PrEP	Cross-sectional survey, online	56 physicians, nurse practitioners, and physician assistants	Planned behavior Diffusion of innovation
	[44]	Determine the practicality of using PrEP for HIV prevention	∙ Medical visit forms ∙ Monthly conference calls with partnering agencies	Administrative cross-sectional	117 men who have sex with men and transgender women	No
	[45]	Evaluate whether insurance status is associated with PrEP utilization	∙ PrEP utilization	Retrospective chart review	201 patients at PrEP clinics	No
	[46]	Explore the role of geosocial networking applications in facilitating PrEP access	∙ Use of geosocial app for HIV testing and PrEP services ∙ PrEP linkage	Retrospective chart review	98 men who have sex with men	No
	[47]	Examine PrEP awareness, familiarity & comfort to prescribe; barriers and facilitators	∙ PrEP awareness/experience/ ∙ Comfort prescribing PrEP	Cross-sectional survey, online	525 physician, nurse practitioners, and physician assistants	Purview Paradox
	[48]	Determine whether healthcare provider contact is associated with PrEP awareness	∙ Awareness of PrEP ∙ PrEP use	Cross-sectional survey	401 HIV-negative people	No
	[49]	Examine awareness of PrEP	∙ Awareness of PrEP	Cross-section population-based survey	118 women who inject drugs	Network theory
2018	[50]	Evaluate HIV PrEP administration and outcomes	∙ HIV exposure risk ∙ STDs, including HIV ∙ Reasons for PrEP discontinuation	Retrospective chart review	159 patients initiating PrEP	No
	[51]	Explore associations between demographics, familiarity, and experience and willingness to provide PrEP	∙ Willingness to prescribe PrEP ∙ Perceived abilities to prescribe PrEP ∙ Patient and pharmacist-level barriers	Cross-sectional survey	140 pharmacists	No
	[52]	Determine association between biases/PrEP clinical decisions & effect of education	∙ PrEP-related clinical decision-making	Cross-section survey, vignette-based	115 medical students	No
	[53]	Assess effectiveness of a one-time secure message or letter to support PrEP care linkage	∙ Linkage to PrEP care ∙ Filing a PrEP prescription	Administrative (clinic) data, prospective	126 patients with a history of STI	No
	[54]	Investigate the association between substance use and PrEP adherence and STI incidence	∙ PrEP adherence	Longitudinal	391 MSM and 3 transgender women in a PrEP clinical trial	No
	[55]	Explore factors that might indicate elements of PrEP-related social control	∙ Partner PrEP use ∙ Willingness to convince partner to initiate PrEP	Cross-sectional (national cohort)	409 MSM in relationships who are not on PrEP	Couples Interdependence
	[56]	Examine PrEP engagement to examine barriers and facilitators at each step	∙ PrEP referral ∙ Patient contact by a PrEP team ∙ Initiating PrEP	Administrative (clinic) data, prospective	785 patients referred for PrEP	No
	[57]	Examine PrEP care outcomes, in particular PrEP retention	∙ PrEP retention	Retrospective chart review	107 patients prescribed PrEP	No
	[58]	Identify factors associated with PrEP dispensing and comfort with PrEP counseling	∙ Pharmacist PrEP awareness, patient counselling and dispensing ∙ Impact of PrEP use	Cross-sectional survey	284 pharmacists	No
	[59]	Assess reasons for PrEP discontinuation	∙ Past PrEP use and current use ∙ Reasons for discontinuation	Longitudinal	197 young men who have sex with men	No
	[60]	Examine PrEP stigma & individual/geospatial factors (ex: neighborhood LGBT stigma)	∙ PrEP stigma and positive attitudes scale ∙ Knowledge/awareness/communication	Longitudinal and multi-level study	620 young men who have sex with men and transgender women	Social Cognitive
	[61]	Assess PrEP knowledge and experience and describe PrEP attitudes and perceptions	∙ PrEP knowledge and awareness ∙ Professional experience with PrEP ∙ Perceptions/attitudes toward PrEP	Cross-sectional survey	347 pharmacists	No
	[62]	Asses the role of interprofessional collaboration in PrEP access	∙ Interprofessional collaboration, training, and provision of PrEP psychoeducation	Baseline from longitudinal survey	285 providers of social and public health services	Socioecological Perspective
	[63]	Understand facilitators and barriers to PrEP uptake	∙ PrEP awareness, use, feasibility ∙ Sexual behavior ∙ Facilitators/barriers	Cross-sectional survey	184 young men who have sex with men	Transtheoretical Model
	[64]	Describe PrEP eligibility, willingness to use PrEP, and ability to access PrEP	∙ PrEP eligibility ∙ Willingness to take PrEP	Cross-sectional survey	138 people who use injection drugs	No
	[65]	Assess if HIV risk behavior mediates cognitive impairment/intent to use PrEP	∙ Intent to use PrEP	Intervention study data	400 people who use drugs	Developed mediation model
	[66]	Explore the distribution of PrEP-providing clinics in the United States	∙ Number of PrEP-eligible MSM in each state and city	Clinic, county, and state-level data	2094 PrEP-providing clinics	No
	[67]	Investigate between race and comfort discussing PrEP with a provider	∙ Interest in learning about and intention to use PrEP ∙ Comfort discussing PrEP	Cross-sectional survey	501 women	PrEP cascade Behavioral Skills
	[68]	Estimate PrEP adherence, factors associated with high adherence, and PrEP discontinuation	∙ PrEP adherence ∙ PrEP discontinuation	Administrative (national) data	1086 veterans	No
	[69]	Examine associations with PrEP awareness/interest & perceived PrEP coercion	∙ PrEP awareness and interest	Cross-sectional survey, online	210 men and women from the general US population	No
	[70]	Assess PrEP activities, perceived barriers, and desired resources among health departments	∙ Role of local health departments in PrEP implementation	Cross-sectional survey, online	56 health department directors	No

**Table 2:** PrEP implementation qualitative

Year	Article	Key elements of research question (s)	Key measures	Research design	Sample (size and type)	Theoretical approach
2011	[71]	Assess incorporation of PrEP into HIV prevention strategy & impact on sexual practices	∙ Barriers to PrEP use as identified by counsellors	Individual patient notes	26 former clinical trial participants	No
2012	[72]	Explore providers’ plan to develop clinical protocols to prescribe, support and monitor PrEP adherence	∙ PrEP knowledge ∙ Cost and capacity to provide PrEP	Individual interviews	22 primary care providers,	Grounded theory
	[73]	Explore the factors surrounding PrEP acceptability	∙ No description of interview guide	Semi-structured individual interviews	24 men who have sex with men and 6 transgender women	Social ecological planned behavior, grounded theory
	[74]	Elicit attitudes about, and preferences for, PrEP services	∙ No description of focus group guide	Focus groups	87 young African American men and women	No
2014	[75]	Understand providers’ attitudes towards PrEP as preventive intervention	∙ Practitioners’ perceived barriers and facilitators to prescribing PrEP	Focus groups	39 HIV providers	No
	[76]	Adapt and use intervention (Life-steps) for high-risk MSM who are prescribed PrEP	∙ Adherence beliefs/barriers & facilitators ∙ Sexual decision-making	Focus groups	39 men who have sex with men	No
	[77]	Investigate men’s healthcare and HIV testing experiences	∙ PrEP knowledge/willingness/beliefs & intentions ∙ Barriers to access	Focus groups and individual interviews	94 male sex workers and MSM	No
2015	[78]	Obtain critical information for the integration of PrEP into treatment	∙ Barrier and facilitators to implementing PrEP/PrEP trials at clinics	Individual interviews	36 medical and counselling service providers	Grounded theory
2016	[79]	Explore trans-specific facilitators and barriers to PrEP acceptability	∙ PrEP knowledge/concerns ∙ Appropriateness of PrEP ∙ Efficacy of PrEP/stigma	Focus groups	30 transgender women	Gender affirmation
2017	[80]	Examine the barriers to PrEP uptake	∙ Perceived PrEP efficacy ∙ Barriers & facilitators to taking PrEP ∙ Feasibility & acceptability	Focus groups	35 men who have sex with men	No
	[81]	Explore PrEP-related risk compensation attitudes among providers with PrEP experience	∙ PrEP attitudes and prescribing intentions ∙ Equitable provision of PrEP	Semi-structured individual interviews	18 PrEP providers	No
	[82]	Explore PrEP knowledge and attitudes, facilitators & barriers, & message preferences	∙ Knowledge and attitudes about PrEP	Focus groups	23 Latina patients; 21 staff	Yes, grounded theory
	[83]	To examine PrEP stigma or stereotypes about PrEP use	∙ PrEP stigma	Individual interviews	160 men who have sex with men	No
	[84]	Understand proximal and distal factors related to PrEP access and adoption	∙ Attitudes towards PrEP, ∙ Barriers to accessing PrEP	Semi-structured individual interviews	20 rural men who have sex with men	No
	[85]	Understand how discomfort in healthcare settings affects PrEP utilization	∙ PrEP knowledge and interest	Online focus groups	24 men who have sex with men	Grounded theory Care continuum
	[86]	Examine the attitudes and knowledge of PrEP	∙ PrEP knowledge and concerns ∙ Comfort discussing PrEP with medical provider	Focus groups	21 transgender men	No
	[87]	Explore themes regarding attitudes toward PrEP	∙ Awareness of PrEP ∙ Sources of PrEP information ∙ Willingness to use PrEP	Semi-structured individual interviews	25 young transgender women	Grounded theory Syndemics
	[88]	Examine use of ART as PrEP and informal use within geosocial applications	∙ Informal use of ART as PrEP ∙ Discussion of such use within geosocial networking applications	Semi-structured individual interviews	39 men who have sex with men	No
	[89]	Describe barriers and facilitators to linkage to prevention services	∙ Implementation processes, outcomes, and infrastructure ∙ Acceptability and sustainability	Structured individual interviews	40 linkage-to-prevention and HIV testing staff	No
	[90]	Assess perceptions of PrEP use and clinical trial participation	∙ Awareness/attitudes toward PrEP ∙ Barriers to PrEP use ∙ Perceived benefits/risks	Focus groups	30 women (15 mother-daughter pairs)	Health belief model
2018	[91]	Understand factors influencing participants’ PrEP use & for dosing schedules	∙ No description of interview guide	Semi-structured individual interviews	37 MSM former participants in PrEP clinical trials	Grounded theory
	[92]	Understand factors influencing PrEP uptake	∙ No interview guide	Social media data (Facebook comments)	76 Facebook users	Grounded theory
	[93]	Make meaning of moral debate surrounding implementation of PrEP	∙ Perceptions about PrEP, perceived PrEP candidates and impacts on sexual behaviour	Focus groups	32 MSM	Social construction
	[94]	Explore how gender affects preventive healthcare seeking, particularly PrEP	∙ Knowledge and acceptance of PrEP (Black MSM) ∙ Attitudes about Black MSM and HIV prevention (community stakeholders)	Interviews and focus groups (ethnographic study)	31 Black MSM (three interviews each) 17 stakeholders	Theories of gender, (not-specified)
	[95]	Identify barriers and facilitators to PrEP	∙ Perceived barriers and facilitators to oral PrEP	Focus groups	18 transgender women	No

**Table 3:** PrEP implementation mixed methods studies (n = 3)

Year	Article	Key elements of research question (s)	Key measures	Research design	Sample (size and type)	Theoretical approach
2017	[96]	Determine feasibility, acceptability, and preliminary efficacy of health recovery program	∙ Intervention feasibility & acceptability ∙ Adherence	Mixed methods; cross-sectional survey data from longitudinal intervention study; and in-depth individual interviews	40 men in treatment for methadone recently initiated PrEP	Information-motivation-behavior
2018	[97]	Identify patients’ physical and psychosocial experiences with injectable PrEP product	∙ PrEP injection pain ∙ Factors motivating persistence and return for study visits/risk perception	Mixed methods; cross-sectional survey data; and individual interviews	40 MSM and transgender women formerly participated in PrEP clinical trial	No
	[98]	Explore reasons why people discontinue PrEP	∙ Reasons for discontinuation	Mixed methods; cross-sectional survey data from online study; longitudinal (18 months and 24 months)	1071 men men who have sex with men	No

## Results

We included a total of 79 articles in this methodological review: [20–98] 51 (65%) quantitative (Table 1) [20–70]; 25 (32%) qualitative (Table 2) [71–95]; and three (4%) mixed-methods (Table 3) [96–98]. Total of percentages exceeds 100 due to rounding up.

### Key Elements of Research Questions and Research Designs

Reflecting our rigorous inclusion/exclusion selection criteria, all articles, regardless of method type, aimed to identify barriers to PrEP implementation. However, some studies had interrelated additional research questions. For example, several qualitative studies not only identified barriers, but described them [77, 84]. Other studies described the processes by which some patients may access PrEP by overcoming identified barriers [88, 95]. Others focused on exploring structural issues that impact specific groups of people confronting disparaging socioeconomic problems (e.g., racism, homophobia, stigma, etc.), the combination of which deter great numbers of people from accessing PrEP [83]. Research questions focused on: (1) patient and service provider preferred modes of PrEP delivery, (2) patient engagement with PrEP including experiences of discontinuation, (3) patient and provider knowledge about and/or attitudes toward recommending and/or prescribing PrEP, (4) behavioral and psychosocial factors influencing PrEP access, implementation, and adherence, and (5) structural disparities (e.g., race, gender, stigma, etc.) in PrEP access and uptake.

### Key Measures

Measures most commonly used evaluated cognitive factors such as PrEP knowledge, attitudes and concerns; a smaller proportion explored perceived barriers to access, utilization, and adherence; and some explored behavioral (e.g., risk compensation) and social/structural (e.g., stigma) factors. The most recently published studies (2018 onwards) explored more complex PrEP decision-making and uptake. For example, reasons for PrEP discontinuation, PrEP-related clinical decision-making, interprofessional collaboration, training, and provision of PrEP psychoeducation, and the role of local health departments in PrEP implementation were explored quantitatively, data of which may lend itself more readily to informing intervention development.

*Quantitative* articles mostly used cross-sectional surveys (n = 35/51, 68%). Other quantitative articles used retrospective chart review, secondary analysis of cross-sectional population-based survey data, intervention study data, prospective and retrospective collection of administrative (clinic) data, and longitudinal study designs. One quantitative study used multiple data sources, combining clinic/administrative data and a cross-sectional patient exit survey.

*Qualitative* articles used a diversity of approaches, including semi-structured individual interviews (n = 10/25, 40.1%) or focus groups alone (n = 11/25, 44.0%); one of these focus groups used innovative online focus group methods. Two studies included both focus groups and individual interviews, one of which used a longitudinal approach, interviewing participants on three different occasions. The remaining qualitative studies involved qualitative analysis of patient notes and qualitative analysis of social media (Facebook) posts.

*Mixed methods* were the minority (n = 3). One article used a cross-sectional survey in combination with semi-structured individual interviews. The other two articles used longitudinal surveys, one in combination with semi-structured individual interviews and the other with online open-ended survey questions.

### Sample Types and Sizes

Of 51 quantitative studies, 31 were conducted with patients, 19 with healthcare providers, and one used data from PrEP clinics. Of 25 qualitative studies, 18 were conducted with patients, five with healthcare providers, and two included both patients and healthcare providers. All three mixed methods studies were conducted with patients.

Sample size for quantitative studies conducted with healthcare providers ranged from 35 to 9023 participants (median: 238), and sample size for quantitative studies conducted with patients ranged from 18 to 2297 participants (median: 205). Across all quantitative studies, the median sample size per year fluctuated from a low of 164 in 2014 to a high of 335 in 2016. Sample size for qualitative studies conducted with healthcare providers ranged from 18 to 39 participants (median: 36), and sample size for qualitative studies conducted with patients ranged from 18 to 160 participants (median: 31). Across all qualitative studies, the median sample size per year fluctuated from a low of 24 in 2012 to a high of 39 in 2014. Mixed methods sample sizes included 40 (two studies) and 1071.

The largest number of studies (n = 25/79, 32%) with patients focused on MSM (n = 20) or MSM combined with transgender women (n = 5). Other key populations included transgender women alone (n = 4) and men (n = 1), people who use drugs (n = 3), and adolescents (n = 1). Five studies were explicitly focused on African American/Black populations, two of which were focused on Black MSM and two of which were focused on Black women. Articles focused on healthcare providers included physicians, infectious disease and other specialists, fellows, residents, in addition to physician assistants, community-based providers, clinician researchers, nurses and nurse practitioners, pharmacists, and medical students. Notably, one study surveyed health department directors.

### Underlying Theoretical Approach

We identified whether or not the research questions pursued in the articles were theoretically framed, and then we identified the theory/concepts used. One-quarter (n = 12/51, 24%) of quantitative articles, under half (n = 12/25, 48%) of qualitative, and one of three mixed methods (n = 1/3, 33%) described a conceptual framework/theoretical approach, including: grounded theory; diffusion of innovation; message framing; theory of planned behavior; purview paradox; network theory; couples interdependence theory; social cognitive theory; transtheoretical model; social ecological approach; gender affirmation; care continuum model; syndemics; health belief model; social constructionism; and information-motivation-behavior theory. One study described choosing variables based on theoretical significance without explicitly mentioning a theory while another described their own conceptual model, and yet another described including theories of gender, health, and sexuality without specifying.

## Discussion

Research on PrEP implementation has been robust and consistent for the past decade. This literature has employed myriad methodologies and has succeeded in identifying important barriers that affect providers, patients, and health care systems. Studies have focused on populations and communities (e.g., MSM, African America, transgender women) most affected by HIV. In so doing, the literature has uncovered structural barriers and systemic hindrances—PrEP stigma, HIV-stigma, homophobia, transphobia, racism—affecting the most vulnerable individuals.

PrEP was approved by the FDA in 2012. It is not surprising that all articles that were included in this methodological review began to appear around 2011. Up to 2011, articles about PrEP had focused on PrEP awareness and attitudes, summaries and discussions about clinical guidelines, models to determine PrEP eligibility, editorials and descriptive commentaries—these articles were excluded from this review. Since 2012, the number of qualitative, quantitative, and mixed methods publications about PrEP implementation has risen in all three categories; with quantitative studies about patients representing the largest increase. Early studies (2012–2015) focused on factors that could facilitate access to PrEP, and thus they were more likely to be about providers’ knowledge, attitudes, and PrEP acceptability as a novel treatment as a prevention strategy. Later studies, particularly those 2018 onwards, have focused largely on the nuances of PrEP decision-making and ongoing engagement with PrEP.

Although the majority of articles included patients and/or used quantitative study designs, the use of particular methods and study samples has differed over time. Early PrEP implementation studies were primarily qualitative, capturing the voices of patients (2011 onwards) and providers (2012 onwards). Quantitative studies selected for this review began to emerge in 2013 and continued steadily at two or more publications per year, with a total of 20 in 2018.

Qualitative research about PrEP implementation has always been in the minority. Early articles unearthed descriptive information, from both patients and providers, which grounded future quantitative research that collected more specific survey data on barriers to PrEP uptake, accessibility, and delivery. Early qualitative studies were formative and used small samples, except for three studies focused on Black MSM, and which used samples involving 87 (74), 94 (77), and 160 (83) participants. Quantitative exploratory studies began to appear in 2013–2014 and the number of publications grew steadily. More sophisticated evaluative [27], comparative [32], and associative [52] research appeared in more recent years (2016–2018). One innovation in qualitative research came about in 2017; one study [85] used an online focus group method in order to examine how discomfort in healthcare settings affects PrEP utilization.

In general, PrEP research has explored cognitive constructs (knowledge, attitude, opinions, concerns, and awareness about PrEP) that are applicable to both patient and provider research participants. Behavioral measures have included, for example, adherence to PrEP among patients and willingness to prescribe PrEP among providers. However, the majority of articles have been exploratory and cross-sectional. Therefore, there is a dearth of longitudinal articles that could capture the fast-paced changes related to PrEP implementation. For example, it is clear that, over time, both patients and providers have developed more awareness, become more knowledgeable, developed better attitudes and fewer concerns about PrEP. Nonetheless, it is not clear, from this literature, whether these changes are sustained over time or the extent to which identified changes influence patient access and adherence to PrEP. Similarly, it is unclear the extent to which cognitive changes among providers may or may not influence providers’ future prescribing behaviors.

The majority of articles about barriers to PrEP implementation aimed to examine cognitive constructs and behaviors of one or another key actor—service providers *or* patients. Studies about patient-level barriers often have small samples and often focus on one population or another. These studies lack the power and/or demographic diversity that would allow for comparisons across different groups of individuals whose degrees of exposure to HIV might differ. For example, the majority of studies in this review involved MSM—yet few were specific to young Black MSM (who are most exposed to HIV) or to other groups often overlooked (e.g., cisgender women). Studies that allow for comparisons across groups are highly needed at this juncture.

There is also a dearth of longitudinal designs that could illuminate trends in PrEP uptake. We identified six longitudinal studies, published in 2017–2018, one of which uses baseline data to assess the role of interprofessional collaboration (IPC) in PrEP access. Though the study uses cross-sectional data, it stands out in that it shows providers of social and public health services having positive attitudes about and thus engaging in IPC with clinicians who can prescribe PrEP. The article suggests that IPC is a promising intervention that should be further studied [62]. One longitudinal article [66] explores past and current use of PrEP and it stands out for its unique contribution about reasons for discontinuation of PrEP. This structural-level quantitative study combines clinic, county, and state-level data to explore the distribution of publicly listed PrEP-providing clinics in the US and to match this distribution with need based on HIV incidence, among other factors. Nonetheless, this study involved only young MSM, and it is thus limited in terms of generalization to other populations. A more recent longitudinal study adds interesting knowledge in that it investigates associations between substance use, PrEP adherence, and the incidence of sexually transmitted infections among MSM in a PrEP clinical trial [54]. The other longitudinal study [60] contributes to knowledge about the role of stigma among a fairly large sample of 620 MSM and transgender women. The unique contribution here is the multi-level approach that includes individual- and geospatial-level data.

The combined longitudinal survey data with geo-spatial city-level data has advanced knowledge about geographic- and individual-level associations with PrEP stigma [60]. Previous articles identifying stigma, as a major factor that influence negatively PrEP uptake, were mostly qualitative and published before 2018. Two key qualitative studies involved a large sample of 160 MSM [83] and of 30 transgender women [79]. In these articles, stigma is studied in relationship to knowledge, and appropriateness of PrEP for specific populations. Articles concerning the role of stigma understandably included historically stigmatized and under-served populations; nonetheless, future research is needed to fully understand the role of PrEP-stigma specifically and how PrEP-stigma manifests in *all* populations in need of HIV prevention. For example, in more recent studies [38, 40], the authors examined the influence of alcohol and drug abuse on patients’ beliefs and whether such beliefs influence PrEP uptake. These and other studies examining associations between alcohol and/or drug use and PrEP access, adherence, and discontinuation could also examine the influence of PrEP-stigma and thus further elucidate current understanding of stigma in the context of PrEP implementation.

Worthy of note is a longitudinal study among the few mixed methods studies in the PrEP implementation literature. We identified three mixed methods studies in 2017 and 2108. One article [96], which was framed by Information-Motivation-Behavior theory, used longitudinal data from an intervention study involving 40 men receiving methadone treatment and who recently initiated PrEP, and also semi-structured individual interviews. The authors of the other two mixed methods articles [97, 98] did not use a theoretical framework, but by using mixed data, they were best able to identify barriers to implementation and reasons for both discontinuation and re-initiation of PrEP. For these reasons, the mixed methods articles stand out among all the others.

### Recommendations for Future PrEP Research

Having described how the methods used to study PrEP implementation have evolved over time, below we provide recommendations about how to improve PrEP implementation research as we move forward. These recommendations concern strategies to improve PrEP implementation research related to (1) designs and methods, (2) the need to involve practitioners in PrEP research and to address discipline shortages, (3) the need to address a lack of attention to the effect of geographic disparities in PrEP implementation; (4) the need for more robust research to address PrEP stigma, and (5) the need for conducting conceptually sound PrEP research.

#### Longitudinal and Mixed Method Research

This methodological review revealed few longitudinal and mixed methods studies. However, we demonstrated that longitudinal and/or mixed methods studies can contribute much to our understanding about how patients and providers may change cognitive structures over time and then change behaviors that may increase or decrease PrEP implementation. This type of longitudinal information is sorely needed so that researchers and policy makers can be best able to develop and test interventions to keep people engaged within what Nunn et al. [99] termed as the nine-step PrEP care continuum—identify individuals exposed to HIV, increase individual HIV-risk awareness, enhance PrEP awareness, facilitate PrEP access, link to PrEP care, prescribe PrEP, initiate PrEP, adhere to PrEP, and retain individuals in PrEP care. Similarly, articles combining qualitative and survey data have been best able to contextualize barriers to implementation and best explain how we might overcome them.

#### Ethnographic and Observational Research

We have been unable to identify more than one ethnographic or observational study that would provide details about the conditions under which patients and providers make PrEP-related decisions, and how, in turn, different decision-making strategies might influence diverse demographic patient groups to access and adhere to PrEP. One article, reporting findings from an ethnographic approach, advances current knowledge by revealing structures, such as the healthcare system and the labor market, which alone or together may hinder PrEP uptake by systematically constraining men’s access to primary providers [94]. More studies that focus on day-to-day functioning of organizations that provide PrEP services are needed in order to advance knowledge about referral-making strategies that might lead to successful access and adherence to PrEP. Such studies could also integrate policy analysis in their designs in an attempt to clarify the influence of policy guidelines on PrEP-related behaviors concerning both patients (e.g., adherence) and providers (e.g., prescribing). For example, in one article in this review [45], the authors showed that insurance status was associated with PrEP use. This important information could be more helpful if the authors had incorporated contextual data to show *how* insurance status influences PrEP use.

#### Involving Practitioners in PrEP Research

In order to help patients to access and adhere to PrEP, providers of social and public health services have a crucial role as they have the knowledge and skills to “move” patients along the PrEP continuum. Regrettably, these providers have been neglected in the current literature. Nonetheless, our research team has shown, for example, that in multivariate analysis higher interprofessional collaboration scores were associated with delivering psychoeducation about PrEP and linking patients to more services along the PrEP continuum [62]. This is an area of research with great potential to uncover specific factors related to interprofessional collaboration and which might inform future intervention for service providers. Therefore, we recommend further inquiry in this area.

#### Geographic Disparities in PrEP Research

Few studies [60, 66] were found that account for the availability of PrEP in specific geographic contexts. Because HIV risk is different within populations in myriad geographic areas, PrEP research will need to focus on these populations and special attention will be needed in order to uncover the specific socioeconomic factors that influence PrEP implementation and how these factors may differ by geographic and political contexts. This level of detail will be needed in order to address disparities related to PrEP access and uptake.

#### Discipline Shortages Need to be a Focus in PrEP Research

Only two articles [82, 94] contained both provider and patient data, and only one study examined data from administrators [70]. We know that structural-level barriers, such as shortage of certain professions in select geographic locations and lack of administrative supervision have an impact on patients’ capacity to access PrEP. For example, it is very hard to find infectious disease physicians, those more likely to prescribe PrEP, in rural areas [100]. Similarly, there are enormous disparities concerning the presence of providers of social and public health services in rural and urban areas, and many may lack regular supervision [101]. A national survey of social workers showed that only 8% of respondents practiced in rural areas [102]. Without the help of competent providers, many patients cannot move through the PrEP continuum and will be exposed to HIV without protection. Therefore, research in this area is encouraged.

#### PrEP Stigma Research

Research to uncover the influence of PrEP-stigma is needed across all populations. We hypothesize that higher degrees of stigma will be found among populations historically under-represented in research (e.g., women and young people), and among racial/ethnic and sexual minorities. Though we have identified articles that examined the role of stigma, future studies ought to examine the intersectional nature of stigma and how it manifests for people facing myriad intersecting structural disadvantages, such as poverty, multiple medical issues, racism, xenophobia, and others. Moreover, since HIV exposure is higher among individuals who use drugs and alcohol [103], we recommend studies to uncover the specific needs of this population.

#### Conceptually Sound PrEP Research

Only 25 out of 79 articles explicitly identified a conceptual framework. The majority of these articles used individual-level theories to guide their choices of variables. These studies did not examine either theoretically or empirically structural issues that may influence PrEP implementation. Compared to articles without conceptual frameworks, those that were framed theoretically were more useful in that they generated findings that can more readily inform the development of interventions for both patients and providers. The same theoretical approaches used to uncover barriers to PrEP can inform intervention development. Moreover, we recommend a combination of theory, longitudinal design, and mixed method approaches. Studies that used this combination allowed the authors to make stronger assertions about their findings and also about corresponding interventions that they recommended.

## Conclusion

Based on our methodological review of the PrEP implementation literature, we conclude that the methods used thus far progressed since PrEP became a major HIV prevention strategy. From a preponderance of formative and descriptive small qualitative studies, we have developed larger and more predictive studies. Nonetheless, there is a paucity of longitudinal and mixed methods studies, those with the best potential to illuminate future practice and policy development regarding PrEP implementation. The majority of studies identified lack theoretical frameworks, and thus may have limitations concerning the applicability of their results. The integration of theory in health services research can improve methodology, which ultimately produces stronger research findings to inform decision-making at organizational and policy levels [104]. As we move forward, researchers will need to strive to take this information into consideration when developing and conducting studies about PrEP implementation. In so doing, we might be better able to develop interventions to break PrEP implementation barriers situated at multiple ecological domains, and thus improve PrEP access, uptake, and adherence. Future research should also shift from models of “cultural competency” to “structural competency” [105] as a new approach to address structural stigma [106] affecting the most vulnerable populations exposed to HIV.
